# Acceptability of an economic support component to reduce early pregnancy and school dropout in Zambia: a qualitative case study

**DOI:** 10.1080/16549716.2019.1685808

**Published:** 2019-11-18

**Authors:** Emmanuel Banda, Joar Svanemyr, Ingvild Fossgard Sandøy, Isabel Goicolea, Joseph Mumba Zulu

**Affiliations:** a Health Promotion and Education Department, University of Zambia, School of Public Health, Lusaka, Zambia; b Centre for International Health (CIH), Department of Global Public Health and Primary Care, University of Bergen, Bergen, Norway; c Center for Intervention Science in Maternal and Child Health (CISMAC), Bergen, Norway; d Umeå International School of Public Health (UISPH), Umeå University, Umeå, Sweden

**Keywords:** Acceptability, Cash transfer, adolescent health, Zambia

## Abstract

**Background**: Cash Transfer (CT) schemes have become popular poverty reducing measures in many low and middle-income countries. Research indicates that when provided to girls in resource poor settings, cash transfers can increase education and postpone marriage and pregnancy. However, a few studies indicate that they can also have negative effects which can affect their acceptability, such as generating intra-community tensions.

**Objective**: Conceptually informed by Rogers’ diffusion of innovation theory, this paper explores factors affecting the acceptability of economic support in a randomized controlled trial in rural Monze and Pemba Districts of Southern Province in Zambia.

**Methods**: Qualitative data were collected through five focus group discussions and six in-depth, semi-structured interviews and analysed using thematic analysis. This study was done in the combined arm of a trial where girls received both economic support and participated in youth clubs offering sexuality and life-skills education.

**Results**: In the study communities, acceptability was encouraging by the belief that economic support provided benefits beyond beneficiaries and that it improved access to education, and reduced teen pregnancies, marriages and school drop-out. However, provision of economic support only to selected girls and their parents and fear among some that the support was linked to satanic practices negatively affected acceptability. These fears were mitigated through community sensitisations.

**Conclusion**: The study demonstrated that relative advantage, observability, simplicity and compatibility were key factors in influencing acceptability of the economic support. However, to enhance acceptability of cash transfer schemes aimed at addressing early marriage and pregnancy, it is important to explore socio-cultural factors that create suspicions and negative perceptions and to provide schemes that are perceived as relatively better than available similar schemes, understood, compatible and viable beyond the immediate beneficiary.

## Background

Despite effects on health and the risk of perpetuating poverty among adolescents, teenage pregnancy continues to be common in many low and middle-income countries (LMICs). This is a public health challenge because girls under the age of 18 may not be mature or emotionally developed enough to have a healthy pregnancy or to give birth [1]. Each year in LMICs, 2.5 million females aged under 16 and 16 million females aged between 15 and 19 give birth and 3.9 million have abortions [1]. In Zambia, 28.5% of girls aged 15–19 years have been pregnant [2]. The rates are higher in rural areas, where 37% report ever being pregnant compared with 20% in urban areas. Those out of school and in the lowest wealth quintiles are more likely to become pregnant than those in school and in the highest quintiles [3]. This suggests that being poor and living in rural areas exposes adolescents to factors and processes that predispose them to early pregnancies. Previous research indicates that adolescent pregnancy in Zambia is often intertwined with and driven by various social, economic and sexual-relations patterns that are further influenced by other underlying issues such as gender dynamics where women and girls are regarded as the weaker sex [4]. Girls often have little choice regarding whether and when to get pregnant and married which undermines their human rights and compromises their opportunity to fully realize their socioeconomic development potential.

Social cash transfer (CT) programmes have shown positive results on adolescent educational attainment and a range of other dimensions, including health, child protection and safe transitions to adulthood in many LMICs [5,6]. They have been used as social safety schemes that provide vulnerable individuals with regular cash payment to lessen deprivation and improve their general well-being as well as improve other aspects of their lives. Social cash transfers are even more vital in LMICs’ fragile settings where shocks occur more frequently and vulnerable households are hit hardest, forcing them to face shortfalls that restrict their capacities to cater for their family needs. Regular transfers tend to alleviate these constraints. CTs in LMICs, like Sub-Sahara Africa (SSA), are specifically configured to respond both to the general needs, such as improving human capital, improving reproductive health outcomes, including STI prevention and forced or early marriage, and the specific needs facing the region, such as food security and survival [7]. In Malawi, Baird and colleagues found that payment of school fees combined with a small cash transfer to adolescent girls and their families resulted in reduced pregnancy rates among girls enrolled in school and led to lower prevalence of HIV and Herpes simplex virus type 2 [5]. The diverse cultural settings in LMICs also play a key role in shaping their delivery and impact that CTs achieve. A study by Dako-Gyeke et al. [8] in Ghana found that although CTs were initially meant for targeted vulnerable children, the transfers were being spent on all the children in the household. This was due to Ghanaian culture whereby all children eat from the same pot and thus those selected as direct beneficiaries shared with other children in the household. CTs have been applied in developed and developing countries and in 2013 nearly 1 billion people were covered by this type of protection [9]. In Zambia, the government has piloted different social cash transfer schemes aimed at cushioning deprivation among selected vulnerable groups since the first Child Grant Program in 2010 [10]. One such scheme is the Keeping Girls in School (KGS) which aims to increase girls’ access to secondary education through payment of school fees and rentals for weekly boarding for eligible girls [11]. The initiative, implemented by the Ministry of General Education, targets 14,000 Secondary School girls (Grades 8–12) from households that have been selected for the government’s social cash transfer schemes for vulnerable families across the country.

Studies on factors that shape the acceptability of social cash transfer schemes aimed at addressing early pregnancy and marriage are very scarce. To the best of the authors’ knowledge, the only related studies were done by Skovdal et al. [12] and Pavanello et al. [13]. Skovdal et al. examined the acceptability and perceived impact of a community-led cash transfer programme in Zimbabwe. They found that community participation, combined with the perceived impact of the cash transfer programme on children’s health and education, led community members to speak enthusiastically about the programme [12]. Pavanello et al. reviewed SCT programmes in Africa and Middle East and found that although cash transfers have positive effects on community bonding, they can also have negative effects, particularly in fuelling intra-community tensions and generating feelings of unfairness, which might lead communities to shun social cash transfer schemes [13].

The current study was done within the Research Initiative to Support the Empowerment of girls (RISE), a cluster randomised controlled trial in Zambia examining the effectiveness of economic support for adolescent girls and their families, alone or in combination with community dialogue meetings, and youth clubs providing sexuality and life-skills education [14]. Prior to the trial, a pilot study was conducted to test the feasibility and acceptability of the different intervention components. It indicated that some communities had reservations over being given free economic support by people they considered strangers, indicating issues related to the acceptability of the intervention [15]. Due to these experiences, the current study aimed to explore more deeply factors influencing acceptability of the economic support in RISE trial in order to contribute to formulation of locally acceptable cash transfer schemes aimed at addressing early pregnancy and marriage in Zambia and elsewhere.

## Theoretical framework

Various theories explain the process of adoption of interventions. The Diffusion of Innovations Theory, developed by [16] is one of the common frameworks for examining the adoption and development of new ideas and new technology and one of the most widely used theories in understanding adoption of innovations [17]. It was therefore chosen as the theoretical framework to examine the acceptability of the economic support in RISE. In its basic form, diffusion is defined as the process through which an innovation is adopted and gains acceptance from individuals or members of a community [18]. Rogers’ Diffusion theory was initially developed to study how new technology and other interventions are diffused ‒ how, why and at what rate they spread. In this case, however, there is no diffusion, but we apply the concepts to study process of acceptability and adoption.

Drawing from the theory of diffusion, an innovation is more likely to be accepted by the adopting system and thus, would be scalable if it has attributes of 1) Relative advantage, which is the degree to which an innovation is perceived as being better than the idea that it supersedes or existing similar programmes; 2) Compatibility or the degree to which an innovation is perceived as consistent with existing values, past experiences and needs of potential adopters; 3) Complexity, which is the degree to which an innovation is perceived to be difficult to understand and implement or access; 4) Trialability pertains to how easy an innovation can be tried on an experimental basis, and finally, 5) Observability is about the degree to which the results of the innovation are visible or viable to others. These elements can help identify factors that could determine whether a target audience may accept economic support to adolescent girls or not.

## Methods

The RISE trial was conducted in 12 districts of Zambia and measured the effects of economic support and community dialogue on adolescent childbearing, early marriage and education [14,19]. RISE has three study arms and recruited approximately 4900 girls enrolled in grade 7 in 157 schools in 2016. We recruited girls in grade 7 as they were about to reach the transition between primary and secondary school when the risk of not continuing in school is high (because of school fees, distances, academic requirements at secondary level) and the risk of getting married and pregnant is high. In the first intervention arm, participating girls were given monthly cash transfers (ZMW30 or about USD 3), while their parents were offered an annual grant (ZMW350 or about 35 USD). A teacher was appointed at each intervention school to manage the distribution of the transfers. In addition, girls’ school fees were covered for girls who enrolled in grades 8 and 9 (junior secondary school) in 2017 and 2018. In the second intervention arm, the same economic support was combined with community meetings and youth clubs for girls and boys to provide education on sexual and reproductive health and life-skills. The third arm was the control arm where none of the above was offered. These interventions were provided from September 2016 up to the end of November 2018, and the girls will be followed up until the end of 2020.

### Study design

This exploratory qualitative study used semi-structured focus group discussions and interviews that allowed us to explore factors influencing acceptability of the economic support in RISE. It was important to allow participants to use their own words to describe their experiences, therefore, we chose to use group discussions and individual interviews. This also gave us the flexibility to explore a subject deeper with the participants. In the focus group, the respondents could also state their agreement or disagreement with the responses and statements of other participants. This elicited the dynamic of interaction of participants debating with one another about different sides of an issue.

### Study location, study participants and sampling

Data were collected from five rural schools and surrounding communities in Monze and Pemba Districts of Southern Province in Zambia. These schools were in the arm receiving both the economic support and community meetings and youth club interventions. Purposive sampling, defined by Cohen et al. (2000) as sampling for a specific purpose and picking a group who fit a profile, was used to select the study participants. To gather a broad range of perspectives, community members with varying degrees of involvement were invited to participate in the study. Pupils were chosen from class registers and community members were chosen with the help of the RISE project team. This paper reports on the perspectives of 24 adults and 16 youths aged around 15. In total, six persons participated in individual interviews and 40 in focus group discussions involving five focus groups: one for non-beneficiary boys in the same class with beneficiary girls, one mixed group of beneficiary girls and non-beneficiary boys in same class, one for beneficiary parents, one for parents of non-beneficiary boys from same class as beneficiary girls and one for beneficiary girls. Individual interviews participants included community gate keepers – two village headmen, two teachers, one PTA Chair and one community member. In total, there were 46 participants, out of which 33 were beneficiaries of economic support and the remaining 13 were non-beneficiaries.

## Data collection and analysis

Interviews were conducted in November 2017, about 14 months into the trial. Excepting one that was carried out in English, all interviews were conducted in the local language, Tonga, by the first author. The interview guides were pre-tested to verify their usefulness. The interview guide explored the perspectives of the respondents on the following topics: how the programme was perceived to impact on local cultures and norms, benefits of CT on the target audience and communities and local barriers to programme success. The individual interviews lasted an average of 40 min, while group interviews took about 60 min. The interviews were translated and transcribed into English and coded using NVivo (Table 1). Data coding involved reading field notes taken during and after each interview, coding, sorting and synthesising transcripts into themes aligned to Rogers’ five perceived attributes (see Table 1). We used priori coding and thematic network analysis. The priori coding approach was selected because we started with a pre-existing theory derived from Roger’s perceived attributes theory as framework for understanding factors affecting the acceptability of the RISE economic support.

**Table 1.** Global theme: factors affecting acceptability of RISE economic support.

Codes	Basic Theme	Organising Themes
Ability to buy lotion, soap and food at school Improve school structures Wide benefits	Wide benefits through freedom in how to use the money Use the support for other school related needs Freedom to use economic support as pocket money	1. Relative advantage
Teen Marriages Teen pregnancies Reduce elopement Behavior changes Reduced sexual activities Bargaining Power School attendance School uniform	Reduced Teen pregnancies and marriages Improved general wellbeing and status Increased school attendance	2. Observability
Financial Empowerment Financial relief Selective beneficiaries Coexist with cultural belief Wrong morals Molding good values	Need and key financial challenges Concern about the exclusion of boys Comparability with key community values and interest RISE cash transfer introducing wrong moral	3. Compatibility
Easily understood Money easily accessed Free money misunderstood Satanism	CT intervention clear and easy to use Satanism fears affecting acceptability	4. Complexity

### Ethics

Ethical approval was granted by the University of Zambia Biomedical Research Ethics Committee (UNZABREC; REF. NO. 066-06-17). Informed and written consent was obtained from all participants. We used pseudonyms throughout the report. The audio recordings were stored on a password-secured computer. Recording and transcription was done by the first author and only he had access to the recordings.

## Results

We identified several factors that influenced the acceptability of the economic support in the RISE Trial. The findings have been grouped around four of Roger’s five attributes: Relative advantage, Compatibility, Observability and Complexity. Interview results indicated that trialability was not a factor in accepting or rejecting the RISE economic support, so this was excluded from the findings.

## Relative advantage

### Wide benefits through freedom in how to use the money

The economic support was generally accepted by most respondents because of its perceived wide benefits beyond the primary girl to include other members of the family. In contrast with other social cash transfer programmes, like the World Vision bicycle scheme, it could be used for school requirements of other family members without disturbing the needs of the beneficiary girl. This sentiment was echoed by both beneficiary and non-beneficiary respondents.

“Apart from looking at school requirements for a RISE beneficiary, we allocated the money among the children that we have. So, the little that we got benefitted everyone in the family” (FGD 003, Beneficiary Female Parent, respondent 5).

The bulk payment of school fees for beneficiary girls was believed to benefit the whole community by providing the school with enough funds to improve school infrastructure, like desks. Schools would usually have less money available for infrastructure because many parents struggled to pay the full school fees.

“The school benefits because once they pay school fees, the school is able to buy things needed to run the school like desks and school books” (FG004, respondent 1 – non-beneficiary boy).

The beneficiary girls liked the cash transfer because they could use it as pocket money to buy food at school, shoes, soap and lotion while others also invested in rearing chickens and goats for income sources.

## Compatibility

### Needs and key financial challenges addressed

Most respondents perceived the economic support to have been directly focused on needs and key financial challenges that they faced concerning accessing education and failure to pay school fees and provide other materials. It was perceived to be in-tune with their poverty realities and this influenced its acceptability.

“Absenteeism is brought about because of our poverty. Some children are not able to pay school fees and are not in school. When that package came it enabled parents buy uniforms and other school requirements and send their children to school” (Senior Teacher: ID001).

### Comparability with key community values and interest

Economic support was generally believed to be consistent with the target population’s values and, in some cases, was believed to mould good values in girls. They believed that it also promoted good behaviour, such as abstaining from sexual relations, among girls because it empowered them to focus on their studies instead of looking for money from men. As most respondents were Christians, this was an important aspect that influenced the acceptability of the RISE support. It was perceived as an ‘upright’ project that came in peace.

“RISE came in peace, it never stopped anyone going to church or encourage to do bad things, it is just here to protect girls to keep themselves so that they can learn and be able to have good futures like being a nurse which you can’t if you don’t get an education (Boy non-beneficiary participant 4, FGD004).

On the negative side, some respondents felt that because the economic support only catered for a small number of girls in grade 7 and their families, it was not compatible with the larger community’s needs and expectations.

“Those with boys complain that their children are also poor, so why were they left out?” (Female Parent Beneficiary Participant 5, FGD001).

Some beneficiary youths felt the project was making their non-beneficiary friends look for money from men so that they also could buy personal items like them. According to them, CTs may have contributed to other girls feeling jealous and taking more risks to get cash.

“They look for men to give them money in exchange for sex so that they also have money like us. This is the bad side I have seen” (Beneficiary girl, respondent 1, FGD005).

Some respondents feared that the girls would start engaging in transactional sex after closure of the project because they were used to receiving money.

There were also some salient community contextual factors that affected acceptability. One such factor was the fear of Satanism, which was described by some respondents as being associated with the apocalyptic prophesies being taught in some churches. A story was spread that anyone who accepted the free cash would join Satanism. This caused stress and stigma for those receiving the support during those initial phases. Because of this, it was reported that in some communities, a few recipients had returned the money after receiving it. However, the majority of girls and parents were reassured through the community meetings that were held prior to the start of the intervention period.

“When it started there were some difficulties, there was backbiting and pointing fingers at each other from some community members about joining Satanism, especially from our friends who were not on RISE” (Beneficiary Female parent 6, FGD002).

## Observability

### Increased school attendance

Many girls receiving the support were reportedly attending school more regularly compared to the previous years. This increase was attributed to the economic support and to some extent the encouragement received from youth clubs and community meetings. With good uniforms, books and monthly CTs, girls were motivated to focus on school. Respondents believed that school drop-out due to poverty was reduced and this influenced the acceptability of the economic support.

“Here the poverty that we have makes it possible for children to drop out of school. Now because of RISE money, all children in this class are in school, no one has dropped because there is help, so we are happy about that” (male beneficiary parent, respondent ID002).

### Reduced teen pregnancies and marriages incidences

Most respondents believed that the economic support was accepted because of its perceived influence on reducing teen pregnancies and early marriages. It was believed that before RISE, the number of pregnancies and marriages for the same grades were higher. Most respondents believed that girls were now spending more time on school and homework, thereby reducing exposure to the risks of unintended pregnancies.

“So, from my own observation, I have noticed that, at least these girls who are in grade 8 (beneficiaries), teen pregnancies are reducing. It’s not like what we see in other schools where there is no RISE” (Senior citizen male beneficiary respondent IDOO2).

They also believed that the economic support had reduced child marriages and elopements. Elopements, which refers to young people running off secretly to be married, were believed to happen when a girl and a boy decided to get married due to lack of financial support to go to school. It was reported that when elopement happened before RISE, parents would let the girl get married and stop school due to poverty. They felt that even if they retrieved her, she would still not complete school. In some instances, this would be an opportunity for some parents to raise money through bride prices. Marriage negotiations could also be initiated to save the honour of the girl and her family.

“If a child receiving support from RISE has been eloped, the parents will follow her and bring her back to school. No one has been followed before RISE support” (Beneficiary parent, female respondent 1, FGD003).

“Because once there is no money, parents would just think of marrying off the girl so that they get bride price. But now with RISE CT we cannot think of that because the money is there for school” (village headman ID003).

## Complexity

The interviews indicated that one of the factors influencing the acceptability of economic support in RISE was that its purpose was understood and easy to access. Intensive community education, which was being done through youth clubs and community meetings, was credited as one of the avenues for creating awareness about the purpose of the economic support among the target communities. The cash was accessed every month by the beneficiaries and no hitch was reported to that effect.

“There is no problem … A teacher goes to get the money from town each month to give the girls, which is very good. The information that you have given us about the purpose of RISE CT for me is enough because you have touched in all angles (Village headman, ID004).

The school fees were paid directly to the school and community meetings were conducted at the school and open to everyone. Limited effort was required to access these components and they were also easily understood.

## Discussion

Informed by the conceptual framework provided by Rogers’ theory about the diffusion of innovations, this study explored factors affecting the acceptability of the economic support in RISE. It investigated whether attributes of the economic support and other contextual factors influenced target audiences’ acceptance or rejection of the economic support. Results suggest that although there were reservations in some target communities during the initial phase of the trial, there was generally a wide acceptance of the economic support. We found that only four of Rogers’ five attributes influenced the acceptability of the economic support: relative advantage, observability, compatibility and complexity.

Relative advantage of the economic support was conceptualised in terms of wide benefits beyond primary beneficiaries, compared to other cash transfer schemes in the target communities. This is consistent with previous studies suggesting that if potential users see no advantage in using the innovation, they will not adopt it [20]. In other words, for new cash transfer schemes to be adopted they must have a clear advantage over existing interventions or no intervention at all. Our findings indicate that this was what most respondents, both beneficiaries and non-beneficiaries, felt about the economic support in RISE. The economic support was regarded as enough for its intended purposes as almost all respondents spoke enthusiastically about how helpful the support was. Considering that these were poor peasant households with frequent budget deficits, small as it may appear, the support made a substantial contribution to their needs and this influenced the acceptability.

Observability was another element that influenced acceptability. When the interviews were conducted, the effectiveness of the interventions on childbearing had not yet been measured, but the respondents believed that the economic support had reduced teen pregnancies, marriages and increased school attendance for the girls. They suggested that the economic support had empowered girls by reducing their economic dependence on men, thereby reducing their sexual activities which could expose them to pregnancies and marriages. This probably reflects that the rate of adoption is greater when there is high visibility of successful use of an innovation [21].

Compatibility, measuring the degree of congruence between an innovation, the target and the setting in which the innovation will be used, was another factor that influenced the acceptability of the economic support. Findings suggest that the support was integrated or at least coexisted well with values, experiences and needs of the target audiences. According to [22], compatibility includes the extent to which the innovation is consistent with what people do or what they have been doing or how they live their lives. The respondents expressed that the economic support was consistent with target audiences’ way of life. For instance, the economic support did not require them to change their values, norms, cultures or day-to-day behaviours. This finding is in line with studies which state that compatibility is an important recurrent feature in innovation acceptance studies [22]. Traditions and social norms that indicated the ‘right and appropriate behaviour’ according to the target audience threatened this compatibility [23]. According to Scott et al., anything that is new and perceived not to support traditional patterns can be regarded as threatening [23]. During the pilot and initial phase of the RISE trial, some community members connected the economic support to Satanism which created a sense of fear and affected their acceptability of the trial (Joseph M Mumba et al., 2018). To reduce the fears, a series of community sensitisation activities were used and appeared to be effective in combatting the rumours.

The element of simplicity of the economic support also contributed to levels of acceptance. Most respondents were informed about the purpose of the economic support during community sensitisations and youth club meetings and the information they received helped them seek clarification that facilitated acceptance of the economic support. This was not surprising considering that SCTs are generally considered easy to implement and simple to understand [24]. Initial community sensitizations were credited as making the community aware of the purpose of the economic support and helping facilitate the adoption process. This is in line with findings from [25] who found that community education is central to the communities’ acceptability of SCT schemes and can have secondary impacts on measurable outcomes. This also corresponds to Rogers’ (1995) ‘knowledge stage’ of the adoption process. He defines it as ‘the mental process through which an individual pass from first hearing about an innovation to final adoption’ [26].

All the respondents who took part in the focus group discussions and interviews were from schools where the economic support was combined with youth clubs and community meetings. We did not conduct any interviews or discussions in schools which only received the economic support. Thus, our findings may have been somewhat influenced by the interactions between potential users and the communities where they exchanged information about the RISE economic support. However, judging from the proportion of participants who collected the cash transfers and from the feedback shared by the RISE cash teachers, the acceptability was also high in schools that did not have youth club meetings and regular community meetings. It is possible that the initial community sensitization meetings were sufficient to achieve acceptance of the economic support as they brought potential adopters together with chiefs, teachers and headmen who are known to play an important role in convincing others to adopt an innovation [27] especially in a rural settings of Zambia where they yield substantial influence.

While the research and practice paradigm known as the diffusion theory offers a set of concepts that can be used to explain acceptability of innovations by individuals, not all these concepts are relevant in the acceptability of SCT support. The findings indicated that trialability did not play much of a role in influencing users to accept or reject the economic support. The only issue which came out is that, after using the money and realising that no one was being initiated or converted into satanism as they thought, most community members ended up perceiving the economic support positively. However, this may not be true in all settings as the diverse cultural contexts in LMICs also play a key role in shaping their delivery and impact that CTs achieve.

One main limitation of the study was the perception from the community that the researcher was part of the implementation team. This may have made the respondents appear to be more positive about the support than they actually were. To mitigate this perception, the researcher continuously reminded the study participants that he was an independent postgraduate student researcher. As part of establishing rapport, the study participants were also shown the letter from the ethics committee at the beginning of each interview which confirmed that the researcher was conducting this study as part of his postgraduate study.

## Conclusion

The findings of this study indicated that the innovation adoption process is multifaceted and dynamic. Contextual factors and attributes of the innovation played an important role in its acceptability. More specifically the study suggests that for future social cash transfer innovations addressing early marriage and pregnancy to be accepted, they need to demonstrate that they are relatively advantageous, simple to understand and access, compatible and viable beyond the immediate beneficiary. Additionally, results indicated that the innovation adoption process depends on communication, and community sensitization may facilitate that process. These findings may be useful for cash transfer planners in modelling acceptability of other social cash transfer schemes.

## Data Availability

The datasets for this study are available from the corresponding author on reasonable request.

## Paper context

Diffusion theory proposes a set of ideas that can be used to understand the acceptability of innovations. However, some of these concepts are not relevant in some SCT support acceptance settings. By identifying the parameters of Rogers’ perceived attributes in SCT support acceptability processes, finding from this study can help understand factors that lead to the failure of SCT schemes and why these factors occur. The study can also help in successful dissemination and implementation of community-based implementation systems.
